# Future climatically suitable areas for bats in South Asia

**DOI:** 10.1002/ece3.11420

**Published:** 2024-05-20

**Authors:** Aditya Srinivasulu, Matt R. K. Zeale, Bhargavi Srinivasulu, Chelmala Srinivasulu, Gareth Jones, Manuela González‐Suárez

**Affiliations:** ^1^ Ecology and Evolutionary Biology, School of Biological Sciences University of Reading Reading UK; ^2^ ZOO Outreach Organization Coimbatore Tamil Nadu India; ^3^ School of Biological Sciences University of Bristol Bristol UK; ^4^ Centre for Biodiversity and Conservation Studies Osmania University Hyderabad Telangana State India; ^5^ Wildlife Biology and Taxonomy Lab, Department of Zoology Osmania University Hyderabad Telangana State India

**Keywords:** bioclimatic variables, chiroptera, climate change, ensemble ecological niche modelling, maximum entropy, suitability hotspots

## Abstract

Climate change majorly impacts biodiversity in diverse regions across the world, including South Asia, a megadiverse area with heterogeneous climatic and vegetation regions. However, climate impacts on bats in this region are not well‐studied, and it is unclear whether climate effects will follow patterns predicted in other regions. We address this by assessing projected near‐future changes in climatically suitable areas for 110 bat species from South Asia. We used ensemble ecological niche modelling with four algorithms (random forests, artificial neural networks, multivariate adaptive regression splines and maximum entropy) to define climatically suitable areas under current conditions (1970–2000). We then extrapolated near future (2041–2060) suitable areas under four projected scenarios (combining two global climate models and two shared socioeconomic pathways, SSP2: middle‐of‐the‐road and SSP5: fossil‐fuelled development). Projected future changes in suitable areas varied across species, with most species predicted to retain most of the current area or lose small amounts. When shifts occurred due to projected climate change, new areas were generally northward of current suitable areas. Suitability hotspots, defined as regions suitable for >30% of species, were generally predicted to become smaller and more fragmented. Overall, climate change in the near future may not lead to dramatic shifts in the distribution of bat species in South Asia, but local hotspots of biodiversity may be lost. Our results offer insight into climate change effects in less studied areas and can inform conservation planning, motivating reappraisals of conservation priorities and strategies for bats in South Asia.

## Graphical abstract

Ecological niche modelling of climatically suitable areas for South Asian bats between current (1970–2000) and near‐future (2041–2060) climate scenarios shows that most species are predicted to retain their current areas or experience small losses, and shifts are projected towards higher latitudes. Although overall distributional changes may not be dramatic, this study highlights potential losses in local biodiversity hotspots, emphasising the need for revised conservation priorities and strategies for bats in South Asia in the face of climate change.

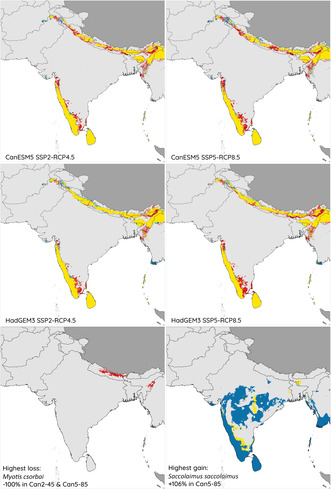

## 1 INTRODUCTION

Climate change is a major threat to biodiversity (Araújo & Rahbek, 2006; Hughes et al., 2003), and its impacts, including the modification of species biology, ecology and distribution and ultimately, increased extinction risk across the world (Parmesan & Yohe, 2003; Schmittner & Galbraith, 2008; Thomas et al., 2004; Walther et al., 2005; Wolkovich et al., 2014), are predicted to accelerate towards the end of the century (Urban, 2015). Loss of species diversity and reduced distribution ranges are expected consequences of climate change (Jetz et al., 2007; Malcolm et al., 2006; Midgley et al., 2006), particularly among taxa with behaviour and lifecycles closely influenced by climatic conditions (Brook, 2009; Sherwin et al., 2013).

Climate change currently affects and will continue to impact many areas of the world including South Asia, which is considered one of the most vulnerable regions to climate change impacts (World Bank Group, 2022). This region hosts a wide and diverse range of biotic and abiotic conditions with spatial variation in climate and vegetation that have resulted in high degrees of diversity, richness and endemism (Srinivasulu & Srinivasulu, 2016) and contains two global biodiversity hotspots: the Eastern Himalayas hotspot and the Western Ghats and Sri Lanka hotspot, and also comprises small parts of the Indo‐Burma, Mountains of Southwest China and Sundaland hotspots (Myers et al., 2000; Olson & Dinerstein, 1998). This biodiversity is likely to be threatened by climate change, but few studies have investigated the potential impacts of future climate scenarios in this region.

South Asia hosts over 500 species of mammals, of which 151 species, in nine families, are bats (Srinivasulu, 2019; Srinivasulu et al., 2023). Unfortunately, in most regions in South Asia, bats are often perceived negatively (Frembgen, 2006) and are not considered to be of conservation value – only six species are specifically protected by the Indian Wildlife (Protection) Act, 1972. Bats can be important as indicator species (Jones et al., 2009), ecological service providers and keystone species (Altringham & McOwat, 2011; Hughes et al., 2012; Kalka et al., 2008; Raman et al., 2023; Williams‐Guillén et al., 2008). Globally bats have been identified as particularly susceptible to climate change (Festa et al., 2022; Sherwin et al., 2013) due to their high risk of dehydration caused by their high surface‐to‐volume ratios (as a result of their relatively smaller bodies and larger wing and tail membranes; Korine et al., 2016; Salinas‐Ramos et al., 2023) and their slower reproductive strategies (Frick et al., 2020). In addition, bat behaviour and ecology are often driven by climate‐based cues (Bates & Harrison, 1997), and due to lacking an effective evaporative cooling body mechanism, bats are especially sensitive to heat (Salinas‐Ramos et al., 2023). Climate extremes like heat waves, increasing in frequency due to anthropogenic climate change (Sippel et al., 2015; Vogel et al., 2019), are known to cause mass mortality events in bats across the world (O'Shea et al., 2016). Overall, bats are likely to be impacted by predicted climate changes in South Asia; however, it remains unclear how changes in climate conditions could affect areas of bat diversity and species distribution ranges in this region, and how these effects vary between species.

Ecological niche modelling (ENM) is a set of techniques widely used to spatially model suitability by extrapolating from ecological niche conditions present within a species' current distribution (Araújo et al., 2006; Pearson & Dawson, 2003). When climate conditions (e.g. temperature and precipitation) are used as niche data, ENM results in climatic suitability envelopes that may approximate the fundamental niche (Soberón & Arroyo‐Peña, 2017). Climatic suitability envelopes are subsequently compared to evaluate changes in climatically suitable locations into the future based on modelled climate scenarios, as an assessment of the effect of climate change on the study species (Guisan & Thuiller, 2005). However, due to limitations including uncertainty in data acquisition and generation, modelling methodology, assumptions of statistical analyses and reproducibility of analytical methods, ENM requires careful consideration and application (Feng, Park, Liang, et al., 2019; Feng, Park, Walker, et al., 2019). This has resulted in the development of various robust statistical applications, algorithms and frameworks for ENM (Araújo & Luoto, 2007; Breiner et al., 2018; Drake, 2014; Elith et al., 2011; Hijmans et al., 2005; Pearson et al., 2006), and a rise in the use of these modelling methods in ecology, conservation and policymaking (Araújo et al., 2019). Recently, ensemble ENMs – where multiple ENM algorithms are used on the same data and their results are combined through various consensus methods – have increased in popularity as a reliable method to account for differences between modelling algorithms in ENM, thereby increasing the robustness and interpretability of these models (Thuiller et al., 2009). Through ecological niche modelling, it is possible to estimate suitable regions based on known species occurrences and climate data for a given time period in a region. Climate impacts on species in a region can then be analysed by comparing these suitability envelopes between current and projected future climate conditions.

In this study, we investigate the predicted impact of climate change on bat species in South Asia using geographic occurrence data and bioclimatic variables describing current climates and four near future (2041–2060) scenarios. We used ensemble ENM and carefully constructed sets of simulated pseudoabsences that incorporate uncertainty in the data and considered biological and environmental factors. The consensus output was then evaluated to characterise changes in the size and location of climatically suitable areas for all studied bats and to identify hotspots of diversity based on climate suitability. These results provide information of value for conservation planning, prioritisation and policymaking.

## 2 METHODS

### 2.1 Study area

**FIGURE 1:**
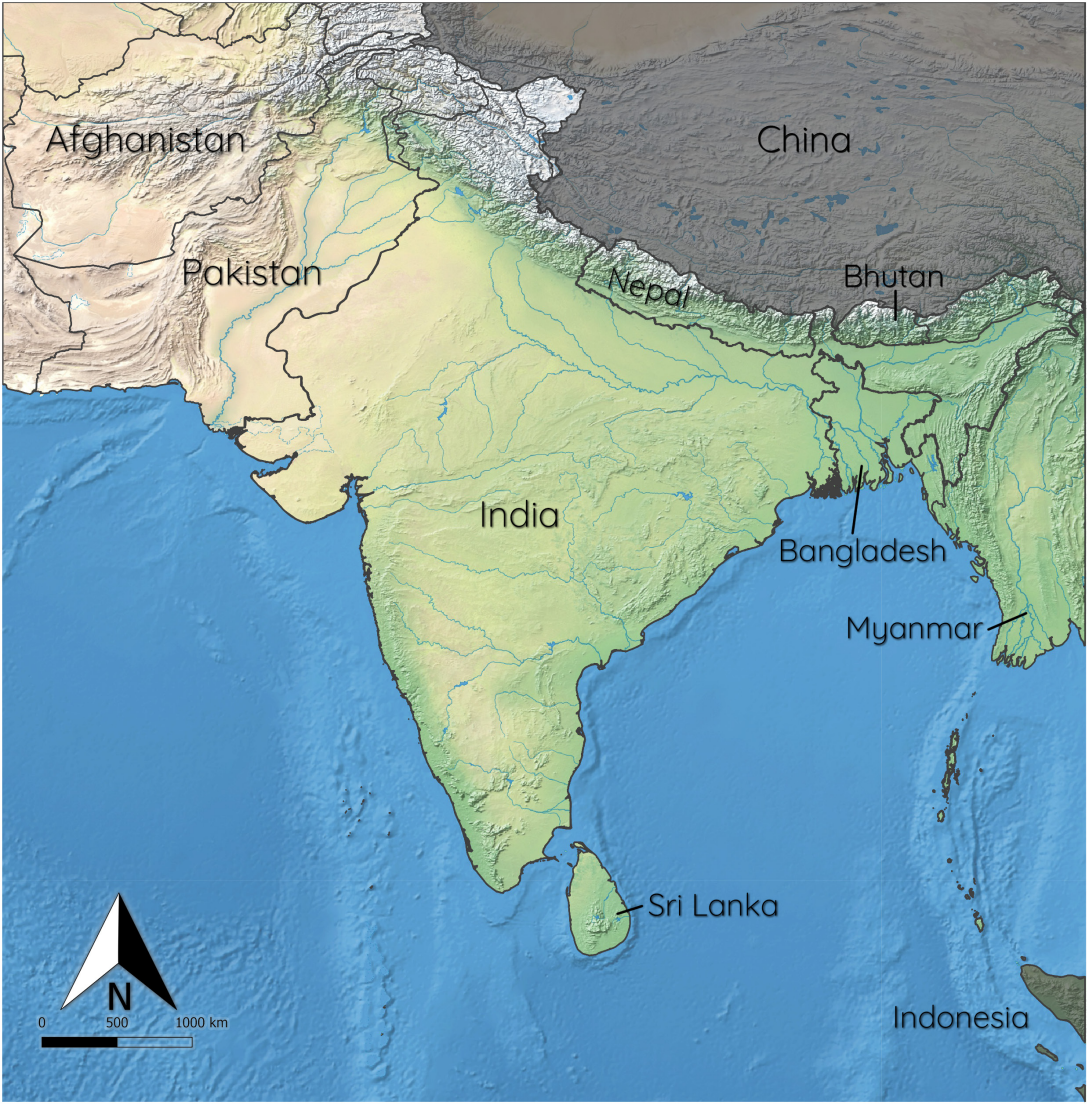
Study area, showing topography and political boundaries of countries in South Asia. Indonesia and China, shown in grey, are not included in this study.

South Asia covers an area of approximately 3.75 million km^2^, and comprises the countries of Afghanistan, Bangladesh, Bhutan, India, the Maldives, Nepal, Pakistan and Sri Lanka (Figure 1). The region consists of four broad climate zones (Oliver, 2005): dry subtropical in the far North, equatorial in the far South, alpine in the mountainous regions and tropical (with regional variations) in most of the rest of the subcontinent. According to the Koppen‐Geiger climate classification system (Peel et al., 2007), the region comprises 15 different climatic subtypes, dominated by subtropical climates (humid summer and dry winter) in the north and the Indo‐Gangetic plain, and tropical savanna climates (wet and dry) in the central, eastern and peninsular regions. Most of the west and north‐west of South Asia consists of arid and desert climates. Due to the large topographical variation in this region, the variety of elevations, soil types and biomes in South Asia is very complex (Ramankutty et al., 2018). To avoid biases based on political boundaries, the focal area for the analysis was defined as a rectangular extent around the borders of South Asia (Figure 1). Due to the Himalayas and trans‐Himalayan regions forming a barrier to bat movement and presence (Ruedi et al., 2008; Thapa et al., 2021), the regions of China that fell under this extent were removed, thus creating a dispersal boundary on the northern border of Nepal, which coincides with the Himalayas. Additionally, due to distance and isolation from most of the study area, the small portion of Indonesia (northern Aceh, Sumatra) that fell within the study extent was removed (Figure 1). The Western Ghats and Sri Lanka biodiversity hotspot is located within the study area, and the area also comprises parts of the Eastern Himalayas, Indo‐Burma, Mountains of Southwest China and Sundaland hotspots (Myers et al., 2000).

### 2.2 Species distribution data

There are 151 recognised bat species in South Asia (Srinivasulu et al., 2023) but we excluded the four species endemic to the Andaman and Nicobar Islands given the geographic isolation of the islands from the rest of the South Asian landmass. Species were identified based on current taxonomic information at the time of analysis (Srinivasulu et al., 2021a, 2021b). We also limited our study to species for which we could gather five or more occurrence localities across South Asia, with a minimum distance between occurrences of 5 km. Presence‐only occurrence data for these species were collected from published (including but not limited to Bates & Harrison, 1997; Raman et al., 2023; Srinivasulu et al., 2021a, 2021b; Srinivasulu & Srinivasulu, 2012), unpublished sources (records collected during field surveys conducted in India between 2002 and 2022 and records communicated by collaborators and citizen scientists in the region confirmed by photographic or other evidence) and GBIF records [accessed July 2022]. Records of specimens housed in museums, including the Natural History Museum (London, UK), Harrison Institute (Sevenoaks, United Kingdom), Field Museum of Natural History (Chicago, USA), Zoological Survey of India (Kolkata, India) and Natural History Museum, Osmania University (Hyderabad, India), were also included.

To define current localities, we omitted occurrence records collected before 1980 and only included records collected between 1980 and 1999 if presence was confirmed during field surveys conducted by the authors from 2000. In order to account for spatial bias in sampling and spatial autocorrelation between occurrences, points were spatially rarefied to the resolution of the climate data (2.5 arc‐minutes). Duplicate records within the same cell were omitted from the analysis using random removal of nearest neighbours implemented in the *spThin* package (Aiello‐Lammens et al., 2015) in R 4.3.0 (R Core Team, 2022). After processing, occurrence data were available for 110 bat species representing all nine families recognised in South Asia, for which we obtained a total of 5998 occurrence points. Data availability varied among species, with occurrences ranging from 5 points for six species (*Coelops frithii*, *Kerivoula lenis*, *K. malpasi*, *Murina leucogaster*, *M. pluvialis* and *Myotis annectans*) to 439 points for *Pteropus medius*.

### 2.3 Bioclimatic variables

Yearly and seasonal patterns in temperature and precipitation, referred to in biogeographic modelling as bioclimatic factors or predictors, are known to influence behaviour and life history in bats around the world (Conenna et al., 2019; Gorman et al., 2021; Kohyt et al., 2021; Weinberg et al., 2022). To capture these conditions and test the hypothesis that macroclimatic effects can determine occurrence of bats, the 19 standard current bioclimatic predictor variables were sourced at 2.5 arc‐minute resolution from the WorldClim v2.1 database for the period of 1970–2000 (Fick & Hijmans, 2017). We focused on this resolution (~4 × 4 km) considering, in the absence of detailed species‐level information, that such distances are within the average foraging range and mobility of these species. Our study covers large taxonomic, spatial and temporal scales that aim to capture broad environmental effects that are best detected using moderately coarse resolutions (Wiens et al., 2009). Finer resolutions are more suitable for detecting smaller‐scale behavioural effects (Pulliam, 2000) including movement, territoriality and inter‐species interactions in mixed colonies.

Four future climate predictions were obtained combining two different global climate models and two different socioeconomic climate scenarios to capture uncertainty. We considered the Canadian Earth System Model 5 (CanESM5; Swart et al., 2019) and Hadley Centre Global Environment Model 3 (HadGEM3; Good, 2019; Good, 2020) available in the Coupled Model Intercomparison Project 6 (CMIP6) from which we obtained 2.5 arc‐minute climate predictions for a near‐future time (2050, averaged from 2041 to 2060). From each model, data were obtained for the shared socioeconomic pathways 2 and 5 (SSP2, equivalent to Representative Concentration Pathway RCP4.5; and SSP5, equivalent to Representative Concentration Pathway RCP8.5). These pathways, used by the International Panel on Climate Change, characterise an optimistic ‘middle of the road’ socioeconomic scenario representing an ideology towards sustainable development, and a pessimistic ‘fossil‐fuelled development’ scenario of climate change based on development almost entirely based on fossil fuels in the future, and little development towards sustainability and an emphasis on resource‐ and energy‐intensive lifestyles (Kriegler et al., 2017). The two CanESM5 models are hereafter referred to as Can2‐45 for SSP2‐RCP4.5 and Can5‐85 for SSP5‐RCP8.5, and the two HadGEM3 models as Had2‐45 for SSP2‐RCP4.5 and Had5‐85 for SSP5‐RCP8.5.

To allow for accuracy in model transfer and clarity in interpretation, bioclimatic variables in ENM models are usually selected a priori to avoid multicollinearity and variance inflation. The bioclimatic variables were first filtered using an assessment of ecological relevance based on field knowledge and literature (Appel et al., 2019; Bates & Harrison, 1997; Corro et al., 2021; Grindal et al., 1992; Stones & Wiebers, 1965). Then, the variables were further filtered based on collinearity – noting that available ENM algorithms are not greatly impacted or can account for correlation and interaction between variables (Dormann et al., 2013; Feng, Park, Liang, et al., 2019; Feng, Park, Walker, et al., 2019; De Marco & Nóbrega, 2018; Muñoz & Felicísimo, 2004), and in some cases, correlated variables may be used if they are considered ecologically relevant. An analysis of multicollinearity was conducted using a combined variance inflation factor (VIF) and pairwise correlation test in the *usdm* package (Naimi et al., 2014) in R. Variables with an absolute pairwise Pearson's *r* < .85 were selected for the analysis. When a variable pair had high correlation (*r* ≥ |.85|), we removed the variable deemed to have least ecological relevance, but if both variables were considered ecologically relevant, the variable with the highest VIF was excluded instead. Additionally, collinearity shifts between current and future predictions were measured using paired t‐tests of VIF scores for each variable, as these tend to impact model transferability (Feng, Park, Liang, et al., 2019; Feng, Park, Walker, et al., 2019). All climate data were cropped and masked to the extent of the study area defined above. A final set of 10 climate variables was selected for the analysis, which were considered ecologically relevant for South Asian bats, and showed no significant collinearity shifts between current and future scenarios (Table S1). The same variables were used for all species for consistent interpretations.

### 2.4 Pseudoabsence generation

As the occurrence data available were presence only, pseudoabsences were generated for use in the ensemble modelling. These pseudoabsences are used by the modelling algorithms as a representation of environmental conditions contrasting those of species occurrences (Elith et al., 2006; Feng, Park, Liang, et al., 2019; Feng, Park, Walker, et al., 2019; Phillips et al., 2006). They are used in presence‐only models as a substitute for ‘true’ absences, where a survey has been conducted for a species, but the species has not been detected. Broadly following recommendations from Barbet‐Massin et al. (2012), 1000 pseudoabsences were specified as the minimum amount to be generated for each species. To balance sample sizes of presences and pseudoabsences for species with few occurrence localities, we generated multiple replicates of pseudoabsences, where each replicate had equal number of presences and pseudoabsences. The number of replicates per species was calculated by dividing 1000 by the number of presences rounded up to the nearest integer. This ensured a minimum of 1000 pseudoabsence points for all species and integrated uncertainty in model results via replication for species with fewer presence localities. Pseudoabsences were generated randomly requiring a minimum distance of 2.5 km and a maximum distance of 1000 km away from occurrence localities. Because data availability varied among species, analyses included from 3 to 200 replicates (Table S2).

### 2.5 Ensemble ecological niche modelling

Presence–pseudoabsence ensemble ecological niche models were generated for each species based on the current climatic data using the BIOMOD framework (Thuiller et al., 2009), implemented in the *biomod2* package (Thuiller et al., 2022) in R. The ensemble included four algorithms known to be robust and perform well across a range of distribution scales (Meller et al., 2014): multivariate adaptive regression splines (MARS), artificial neural networks (ANN), random forests (RF) and maximum entropy (MAXENT, implemented using *maxnet*; Phillips et al., 2017). This diversity in computational models provides low inter‐correlation in model components, which is ideal for higher predictive performance in ensemble ENMs (Elith, 2019; Valavi et al., 2022). The models were calibrated according to the default parameters and parameterisation processes in *biomod2*.

To validate model results, we used fivefold cross‐validation, dividing the occurrence data into five subsets, and testing each subset against a model calibrated on the remaining four subsets. Hold‐out validation, where a small subset of the data is used only for evaluation, while the remaining data are used for calibration validation, was unsuitable due to the small sample size of occurrences in several species. Model performance was evaluated using two standard evaluation metrics: the area under the receiver operating characteristic (ROC) curve (AUC) and the true skill statistic (TSS). Variable contributions were calculated by performing five permutations for each algorithm, each involving removal of the focal variable from the model to calculate the difference in performance. We report the average permutation importance.

The individual models with a final TSS value >0.7 were then combined into ensemble consensus models for each species which we used with future climate data to predict the future climatically suitable areas. Predicted suitability was reclassified into binary suitable–unsuitable predictions using the threshold at which TSS was maximised (values above this threshold represent climatic suitability). These binary maps were used to characterise changes in climatically suitable areas from current to future climatic scenarios using four metrics: the relative change in climatically suitable areas (% change), the percentage of the current area retained into the future and the distance and azimuth (angle of direction) between centroids of current and future suitable areas. Change and retention were calculated respectively as:

$$\text{Change}=\frac{\text{Current suitable area}-\text{Future suitable area}}{\text{Current suitable area}}*100$$

$$\text{Retention}=\frac{\text{Current suitable area}\wedge \text{Future suitable area}}{\text{Current suitable area}}*100$$

To estimate distance and azimuth, we first defined distinct fragments within suitable areas. Fragments represent connected suitable cells from the binary maps, estimated by first applying morphological dilation using a buffer of radius 2.5 km, followed by a morphological erosion using a negative buffer of the same radius. As a result of this process, suitable areas within 2.5 km of each other were connected to reflect the assumption that bats can move easily within this distance. The resulting number of fragments varied among species depending on the configuration of the suitable area. For all fragments, we then identified centroids and the shortest straight paths between each centroid in the current scenario and its nearest neighbour in the future scenario, assuming zero movement costs. The length and azimuth of that path were used to estimate distances and angles of direction. For each species, we calculated the mean distance and mean azimuth. Azimuths were classified into cardinal and intercardinal directions based on angle, assuming North to be 0 and 360. All centroid‐based calculations were performed using the *geosphere* package (Hijmans, 2022) in R.

Summary models of climatically suitable areas were generated for all species together by creating a map of species richness for each time period. All binary models for each time period were summed together into a model that ranged from 0 to 110, representing the number of species for which a cell is climatically suitable according to their binary models. This model was then rescaled to a 0 to 1 scale for consistent comparison and converted into a binary map using a threshold of 0.3, such that in the final binary models for current and future, the positive cells represented suitability hotspots – areas that were climatically suitable for ≥30% (more than 33) of the study species. Certainty in projected models was calculated as a continuous model by first averaging individual replicates within a species to assess model agreement, and then averaging these species models into a final model ranging from 0 to 1, which can be interpreted as high certainty of climatic unsuitability through uncertainty to high certainty of climatic suitability.

## 3 RESULTS

### 3.1 Impact of future climate change

**FIGURE 2:**
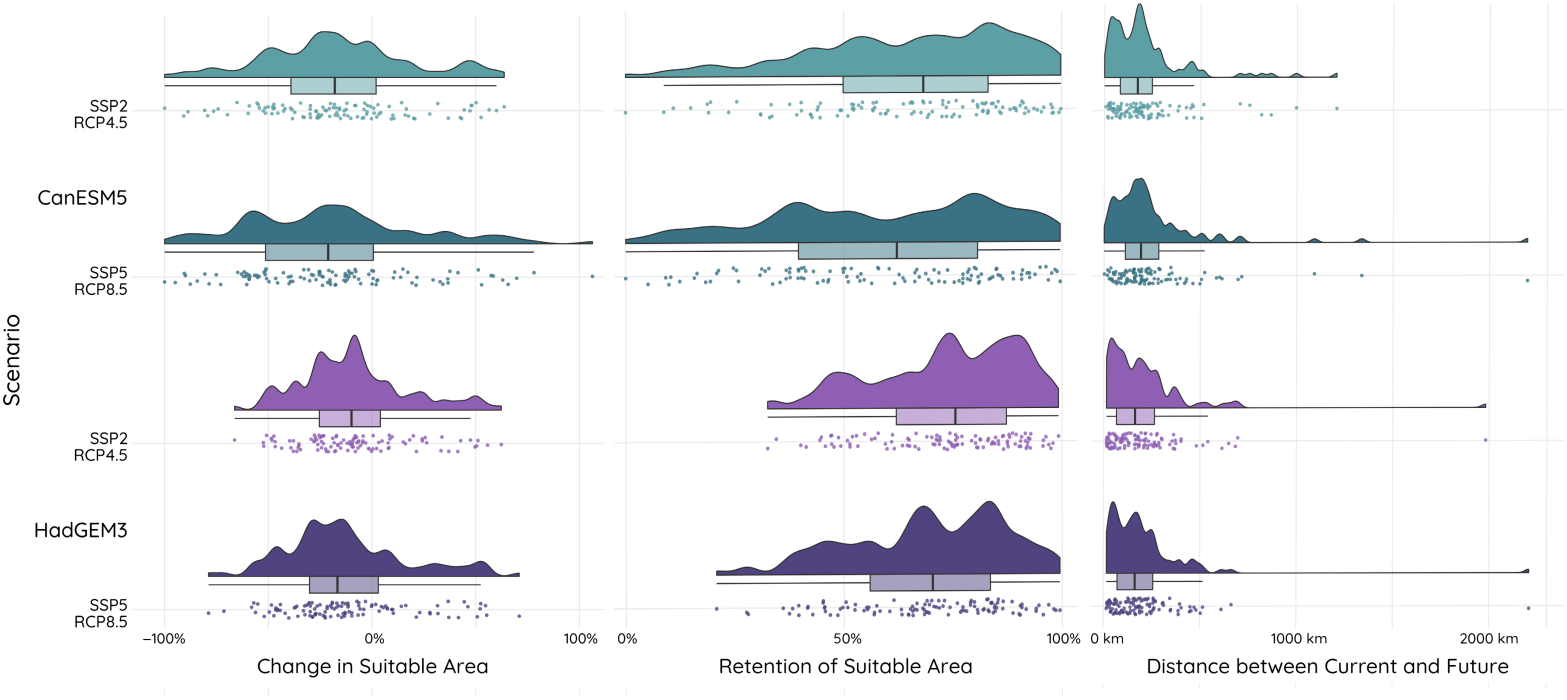
Projected impacts of climate change by mid‐21st century for 110 species of bats in South Asia under four future scenarios representing two climate models and two socioeconomic pathways. Panels show the projected percentage change in climatically suitable area from current to future, percentage of the current climatically suitable area predicted to be retained in the future and distance between current and future climatically suitable areas.

The expected climatic conditions by the mid‐21st century are likely to result in smaller climatically suitable areas for bats in South Asia (average reduction >8%) but with large portions of the current areas expected to remain suitable (average retention >59%. Figure 2). However, these average impacts hide considerable variation among species resulting partly from different climatic variables being important across species and models (Table S1). In all scenarios, there were potential winners and losers, with some species predicted to have no climatically suitable areas in the future while for others the area could double, and retention varied from nearly 0% to 99.8% (Figure 2). These ‘losing’ and ‘winning’ species were more common in some taxonomic groups, with species in the family Miniopteridae being consistently among the highest losers, and species in Rhinopomatidae and Pteropodidae predicted to be among the most affected in the CanESM5 model scenarios and the HadGEM3 model scenarios respectively. On the other hand, Molossidae and Emballonuridae showed the highest gain in suitable areas under the CanESM5 model and the HadGEM3 scenarios respectively. Nevertheless, in nearly all scenarios, even winning families generally had species projected to lose suitable area (Table S2). In the CanESM5 scenarios, Molossidae showed the highest projected retention of suitable areas in the future for any family (73.5% in Can2‐45 and 71.2% in Can5‐85), while Hipposideridae was projected to retain the highest amount of suitable area in the HadGEM3 scenarios (80.7% in Had2‐45 and 76.8% in Had5‐85; Figure 2; Table S2).

Overall patterns were largely consistent among the four future climate change scenarios explored, with smaller impacts predicted under ‘middle of the road’ SSP2‐RCP4.5 socioeconomic scenarios than in the pessimistic ‘fossil‐fuelled development’ SSP5‐RCP8.5 scenarios (Figure 2). Many species show consistent losses, like *Kerivoula malpasi*, known from five localities, with predicted losses of 66% to 84% in climatically suitable areas across scenarios; while others showed consistent gains, including *Myotis hasseltii*, that were projected to increase the area of suitable climate by more than 60% and *Saccolaimus saccolaimus* which is projected to gain 106% of its current suitable area (Figure 2; Table S2). For some individual species, projected impacts were highly dependent on the scenario – for example, *Myotis csorbai*, known from seven localities, was projected to lose 100% of climatically suitable areas in the future in CanESM5 scenarios, but had losses of around 47% in the HadGEM3 scenarios. *Pteropus medius*, with 439 occurrences, and *Cynopterus sphinx*, with 312 occurrences showed moderate projected retention of suitable areas (25.9%–57.1% in *P. medius* and 30.8%–44.8% in *C. sphinx*; Table S2), with varied degrees of projected losses in all scenarios (18%–61.7% in *P. medius* and 37.9%–60.7% in *C. sphinx*; Table S2).

**FIGURE 3:**
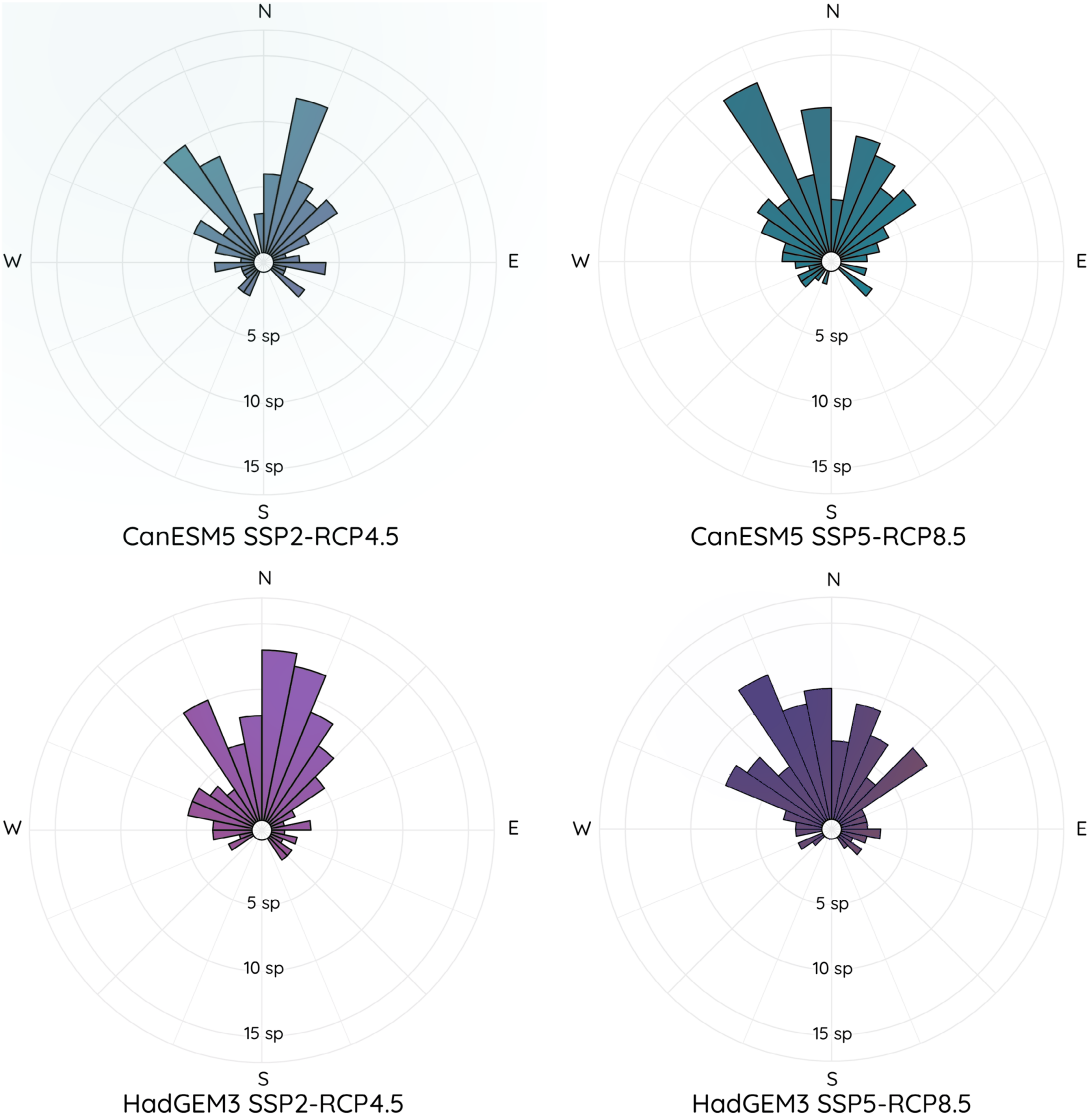
Projected shifts in the location of climatically suitable areas of 110 bats in South Asia caused by predicted climate change by the mid‐21st century under four climate change scenarios representing two climate models and two socioeconomic pathways.

Across all climate scenarios and species, and including all projected spatial changes, future climatically suitable areas were on average 216 km from current climatically suitable areas. However, distances also varied between scenarios and species. For example, *Murina pluvialis*' climatic suitable area was projected to shift by an average of 4.5 km in the future, the smallest distance in the CanESM5 scenarios; however, in the HadGEM3 scenarios, the average distance between current and future areas was 128 km (Table S2). For some species, the disparity in projected suitable areas between socioeconomic scenarios led to extremely large differences in distance between current and future. For example, *Rhinolophus subbadius*, in the SSP2‐RCP4.5 scenarios for both climate models, had a projected distance of 28 km from current to future; however, in SSP5‐RCP8.5, the distance increased to 2202 km. Most future suitable areas were located northwards from currently suitable areas with a trend for more north‐eastern shifts under SSP2‐RCP4.5 socioeconomic scenarios and more north‐western shifts under the more pessimistic SSP5‐RCP8.5 scenarios (Figure 3; Table S2).

### 3.2 Suitability hotspots

**FIGURE 4:**
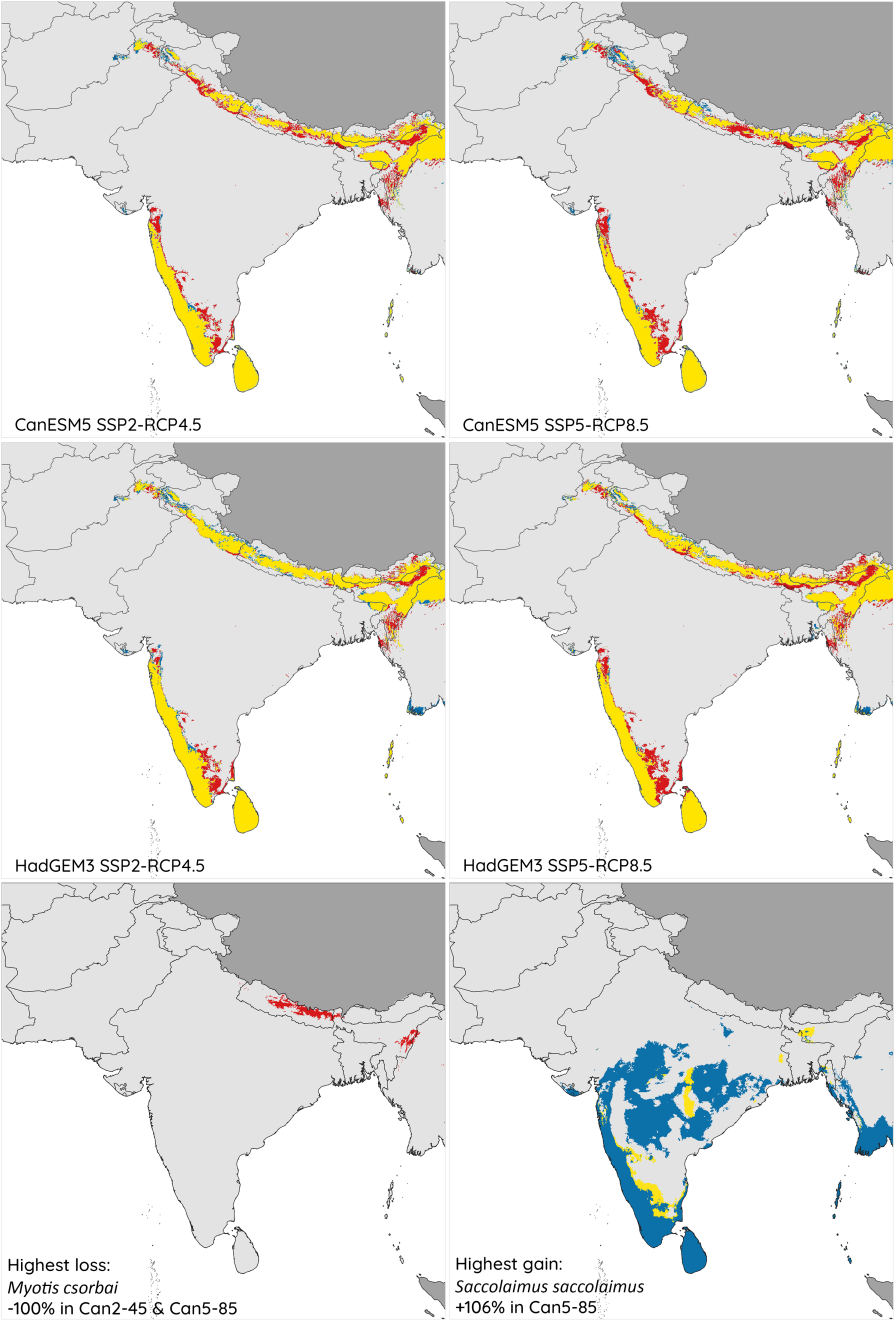
Projected impacts of climate change by mid‐21st century to current hotspots of climatic suitability for 110 bats in South Asia (hotspots reflect areas suitable for >30% of studied species) under four climate change scenarios representing two climate models and two socioeconomic pathways, with maps showing species with the highest loss and gain for any species in any scenario. Current climatically suitable areas are represented in yellow and red, with yellow showing areas projected to remain as climatically suitable hotpots in the future and red representing areas lost. Blue areas are gains projected as new future hotspots (areas currently not identified as hotspots but predicted to be suitable for many species in the future).

The most suitable areas under current climate conditions were projected to host up to 64 of the 110 study species, with 11.9% of the total study area (446,686 km^2^) projected to be suitable for at least 30% of the study species (i.e. 33 species; Figure 4). We detected four contiguous hotspots in all climate models: the lower Himalayas of north and northeast India, northeastern Pakistan, Nepal, northern Myanmar and Bangladesh; the Andaman and Nicobar islands and southernmost coast of Myanmar; Sri Lanka, covering the entirety of the island; and south India, in the Western Ghats and along the coasts of central and southern Maharashtra, Karnataka and Kerala, and fragmented regions of southern Karnataka and southern Tamil Nadu, extending to the southern Nilgiris and Coromandel coast of Tamil Nadu. Additional smaller hotspots appear in the Ballari–Vijayapura–Hubli region of Karnataka, India, an area characterised by unique geography and isolated geology and climate; the highlands west of the Indus Valley in northern Pakistan and eastern Afghanistan; and regions including and immediately north of Gir National Park, southern Gujarat, India (Figure 4).

Most of these hotspots were projected to remain climatically suitable for many species by the mid‐21st century in all scenarios, although their extent was generally reduced, particularly in the lower Himalayas and the Western Ghats hotspots, and projections suggested northward shifts in the Himalayan regions (Figure 4). In northern India and northern Pakistan, projected changes reduced contiguity in projected suitability hotspots (particularly under the CanESM5 scenarios). In the Western Ghats, declines particularly affected central Maharashtra and Tamil Nadu. Additionally, the small distinct hotspots in Ballari–Vijayapura–Hubli were projected to disappear, while hotspots west of the Indus Valley and north of Gir were projected to expand (Figure 4). Although both climate change models predicted similar changes, under both HadGEM3 scenarios, a marked northward increase in suitable areas in the Ayeyarwady region of Myanmar was projected. Analysis of model certainty showed a moderate degree of uncertainty around the projected suitability hotspots in all scenarios, combined with some areas of high certainty of suitability in the Himalayas and northeastern India, and in southern India and central‐southwestern Sri Lanka (Figure S1).

### 3.3 Model evaluation

We found variation in performance of the four algorithms used, with some models having TSS < 0.7 for several species. RF was utilised for all 110 species, while MARS, MAXENT and ANN were limited to 93, 90 and 89 species respectively. For 17 species, RF was the only algorithm represented in the final ensemble. There was a significant negative correlation between the number of occurrence points and the number of models used in the final ensemble (Pearson's *r* = −.791; *p* < .001). RF was the only model represented in the final ensemble for species with more than 120 occurrences; *Taphozous nudiventris* and *Sphaerias blanfordi* were the two exceptions to this, with 97 and 17 occurrences respectively. RF models also had the highest mean AUC and TSS scores across all species (Table 1).

**TABLE 1:** TSS and AUC model evaluation scores for each modelling algorithm used in the ensemble ENM.

Modelling algorithm	TSS	AUC
ANN	0.869 ± 0.059 [0.750–0.999]	0.955 ± 0.024 [0.887–1.000]
MARS	0.897 ± 0.055 [0.761–0.995]	0.970 ± 0.016 [0.920–0.997]
MAXENT	0.859 ± 0.052 [0.754–0.978]	0.958 ± 0.019 [0.904–0.994]
RF	0.980 ± 0.014 [0.940–1.000]	0.998 ± 0.001 [0.991–1.000]
Ensemble	0.879 ± 0.058 [0.726–0.996]	0.976 ± 0.012 [0.932–0.999]

*Note*: Scores are given as mean ± standard deviation [minimum – maximum].

## 4 DISCUSSION

Our analyses of the effects of projected near‐future climate change on bats in South Asia show a general pattern of moderate potential loss in all scenarios with relatively high degrees of retention in climatically suitable areas. As expected, smaller losses were projected under the ‘middle‐of‐the‐road’ SSP2‐RCP4.5 scenarios compared to the more pessimistic ‘fossil‐fuelled development’ SSP5‐RCP8.5 scenarios, reinforcing the need for changing behaviour and avoiding business as usual (Peters et al., 2013). While loss was moderate, our results also revealed variability among species and scenarios with winners and losers. For example, *Myotis csorbai*, a species restricted to Nepal, was projected to lose all its current suitable area in some scenarios, while *Saccolaimus saccolaimus*, a species with a very wide but fragmented distribution in South and Southeast Asia, was projected to double its current suitable area in some scenarios. Previous work has also reported varied responses of bats to climate change. Studies across the world have reported negative impacts both globally (Bellard et al., 2013) and regionally (Rebelo et al., 2010, in Europe; Hughes et al., 2012, in Southeast Asia; Thapa et al., 2021, in Nepal). Bandara et al. (2022) found no or limited effects for *Kerivoula picta* and *K. malpasi* in Sri Lanka. Positive effects have also been observed in the Amazon (Costa et al., 2018), North America (Hayes & Piaggio, 2018) and Nepal (Thapa et al., 2021). This variation likely reflects differences in niche plasticity, robustness to changing conditions and migratory propensity among species. Additionally, variation can occur due to data limitations and modelling choices. Indeed, our results showed uncertainty in projections for some species: many species involved small (< 30 occurrences) sample sizes, and the minimum sample size of five occurrences has been used before but may not be fully reliable (Pearson et al., 2006). However, most advanced ENM algorithms, especially techniques such as MAXENT, can account for fewer occurrences in species with low prevalence and narrow ranges (Morales et al., 2017; Pearson et al., 2006; van Proosdij et al., 2016), and ensemble modelling can provide higher predictive accuracy by accounting for inter‐model variation. Capturing and reporting this variation by considering various scenarios, scales and algorithms is important to ensure that conservation and management recommendations are not misguided due to over‐ or under‐fitting and a lack of reliability in results.

Similar to other studies on bats (in Europe by Rebelo et al., 2010; in Southeast Asia by Hughes et al., 2012; and in Nepal by Thapa et al., 2021), we also found a trend for likely shifts towards higher latitudes in climatically suitable areas. In SSP2‐45 scenarios, suitable areas for most species were projected to be located north‐northeast or northwest from current areas; in SSP5‐85 scenarios, the shift was more generally northeast. Overall, climate change is expected to lead to latitudinal shifts towards polar regions, but arguably, our projected shifts could also reflect the geographic configuration in our study area, where the Southern region is mostly coastal. However, this is unlikely the reason for the observed trend, as for species currently in northern and central areas, we also did not generally find projected southward shifts. Future suitable areas were also usually not located far from current suitable areas, and it is likely that bats could track changes in climatic suitability in many cases. However, there are several barriers that could limit shifts, including the Himalayas in the north and northeast, the Thar Desert and the Great Rann of Kutch, and the hill ranges of south and central India. Similarly, moving across large tracts of water may prevent tracking of climatically suitable areas in some species. Importantly, even if there are no strong geographical barriers, movement may be prevented due to lack of other abiotic and biotic resources or variation in dispersal behaviour.

Combining information from individual species, we identified current and future climatic suitability hotspots – areas of climatic suitability for >30% of study species. The current suitability hotspots aligned with four biodiversity hotspots falling within the region (Myers et al., 2000), but representation varied. While a large proportion of the current suitability hotspots were projected to be retained into the future, projected losses outweighed areas of projected gain. In the Himalaya and Indo‐Burma hotspots, projected losses would lead to high fragmentation and isolation between hotspots (especially under the SSP2‐RCP4.5 scenarios). Movement corridors and continuous protected area networks are likely to be critical to allow movement and occupation of new suitable areas. Interestingly, new suitable areas were consistently identified nearby rivers, around the northern regions of the Indus valley, northwestern Pakistan and southern Gujarat. The small areas of suitability in Ayeyarwady (Myanmar) were projected to increase only in the HadGEM3 scenarios, and in Had5‐85, there were a few additional isolated fragments of projected gain in suitability in Bangladesh and northeast India, regions with typically moist deciduous and wet evergreen habitats (Champion & Seth, 1968; Olson et al., 2001), quite similarly to Ayeyarwady. The Western Ghats suitability hotspot was mostly retained and contiguous across all scenarios, and gains in climatically suitable areas were restricted to small areas on the margins, showing that the Western Ghats are likely to remain stable as a suitability hotspot into the near future. Losses in climatically suitable areas were consistently seen in the Nilgiris hills and northern Maharashtra – both regions with unique vegetation and habitat structures, ranging from semi‐arid scrublands in the north of the Western Ghats to tropical moist deciduous forests in the Nilgiris. Many regions in peninsular India which are known to be specific in geography and habitat consistently showed patterns of projected loss in suitable areas. Combined with a growing understanding of the effects of climate change in the Himalayas and Western Ghats and how it impacts biodiversity (Srinivasulu et al., 2021a, 2021b; Thapa et al., 2021), our results showing high degrees of retention combined with severe losses and minimal gains further support the importance of these regions.

We evaluated projected impacts of climate change using ensemble ecological niche modelling, an approach with good predictive accuracy that captures uncertainty from algorithm choice (Hao et al., 2020). However, this method has some limitations. First, ensemble ENM requires considering the balance between selecting many algorithms and the increased computational time and resources. Generally, the suggestion is that if a smaller number of algorithms are used, the ones with higher predictive accuracy and robustness are selected, as done here (Drake, 2014). A second limitation is the challenge of generating suitable pseudoabsences for presence‐only occurrence data (Barbet‐Massin et al., 2012; Engler et al., 2004; Lütolf et al., 2006). Our method for generating pseudoabsences incorporates random sampling within geographic limits based on spatial resolution and scale of the analysis and estimated foraging distances of study species. We aimed to balance statistical rigour and ecological realism while working with the constraints of lacking accurate species‐level data on bat ranging and movement in South Asia. Further analyses for species with available data could incorporate information on ecological distance and environmental profiling as additional limiting factors to pseudoabsence generation (Iturbide et al., 2015). Model certainty is also an issue with ENM that greatly impacts the reliability and interpretability of these results. For instance, our analysis showed high certainty of climatic unsuitability in most of the study areas (Figure S1). However, it is important to note that per‐species results are much more informative, and broad interpretations must be very cautious. Finally, another limitation of ENM is the need to carefully select variables to balance ecological importance, methodological constraints, including the effects that multicollinearity and collinearity shift have on different algorithms, and model transferability (Feng, Park, Liang, et al., 2019; Feng, Park, Walker, et al., 2019). Here, we also considered this balance selecting robust methods, reducing variables to avoid high collinearity while prioritising ecologically relevant information.

Understanding species responses to rapid climate change is vital in conservation planning, especially in regions with high biodiversity and rates of endemism (Quintero & Wiens, 2013; Raman et al., 2023; Warren et al., 2008). This study is an initial assessment of potential effects of near‐future (2041–2060) climate change on bat species in South Asia, finding that while climatically suitable areas may be reduced, many currently suitable areas are likely to remain, and shifts may be within the dispersal potential of many species. Nonetheless, retention of suitable areas and moderate loss does not necessarily ensure population persistence. Our initial assessment of impacts focuses on abiotic climate effects, but climate change may influence habitat and food resources differently, resulting in climatically suitable areas being effectively unable to support healthy bat populations. Analysing climate effects alone ignores the potential combined effects of climatic and ecogeographic impacts on species distributions (Newbold, 2018). This is, however, a first step to understanding potential changes in areas where climate and ecogeographic data may not be of the same quality, interpretability or accessibility, biasing ecological interpretations. Future climatically suitable areas may also be unsuitable due to geology and topography, factors linked to human activities including human land use, human population densities and proximity of roosts and foraging sites to human infrastructure and developed areas. However, while it is possible to model both types of factors together (e.g. Hughes et al., 2012; Raman et al., 2023; Simões & Peterson, 2018), the lack of accurate data on their interactions when it comes to bats in this region could lead to overfitting, erroneous predictions, and misinterpretations across highly diverse species and functional groups (Araujo & New, 2007; Fordham et al., 2012; Newbold, 2018; Peterson et al., 2011; Simões & Peterson, 2018). It is important to analyse the effects of climate (and future climate change) and ecogeography independently as well as together, and to study their interactions, to truly understand the factors influencing abiotic and biotic ecological suitability for each species. In addition, it is important to consider that ecological niche modelling based on data from currently occupied areas is a limited perspective on the true niche of the species, and may not reflect the species' entire climatic limits or its capacity to adapt to changing environments (Hoffmann & Sgrò, 2011). Future work that considers these additional factors and limitations, and the current intactness of future suitable areas would be important to inform conservation actions for climate change mitigation. Our results offer a first evaluation that highlights the need to further study climate change impacts in megadiverse regions such as South Asia and to develop robust conservation plans that integrate this information. Effective conservation requires the integrated study of species responses to climate, biotic, geographic and anthropogenic factors, and effective communication with and outreach to policymakers and stakeholders at all levels.

## AUTHOR CONTRIBUTIONS

**Aditya Srinivasulu:** Conceptualization (lead); data curation (equal); formal analysis (lead); methodology (equal); project administration (equal); visualization (lead); writing – original draft (lead); writing – review and editing (supporting). **Matt R. K. Zeale:** Conceptualization (equal); data curation (equal); formal analysis (supporting); investigation (supporting); methodology (equal); writing – review and editing (supporting). **Bhargavi Srinivasulu:** Conceptualization (supporting); data curation (equal); formal analysis (equal); project administration (supporting); writing – review and editing (supporting). **Chelmala Srinivasulu:** Conceptualization (equal); data curation (supporting); formal analysis (supporting); methodology (supporting); project administration (supporting); supervision (supporting); writing – original draft (supporting); writing – review and editing (equal). **Gareth Jones:** Conceptualization (equal); formal analysis (equal); methodology (supporting); supervision (supporting); writing – review and editing (equal). **Manuela González‐Suárez:** Conceptualization (equal); formal analysis (equal); methodology (equal); project administration (equal); resources (lead); supervision (lead); validation (equal); visualization (equal); writing – original draft (equal); writing – review and editing (equal).

## CONFLICT OF INTEREST STATEMENT

The authors declare no conflict of interest.

## Supporting information


Figure S1.

Table S1.

Table S2.

## Data Availability

Data used in this study are available on Dryad: https://datadryad.org/stash/share/83WgULbUmh_tIoXiSpo_kC5gVz3OqEy16PlQFUv4H4M.
