# Supplementary figures and images for: Future climatically suitable areas for bats in South Asia
Source: Ecol Evol. 2024 May 20;14(5):e11420. doi: 10.1002/ece3.11420 (PMC11106050; doi:10.1002/ece3.11420)

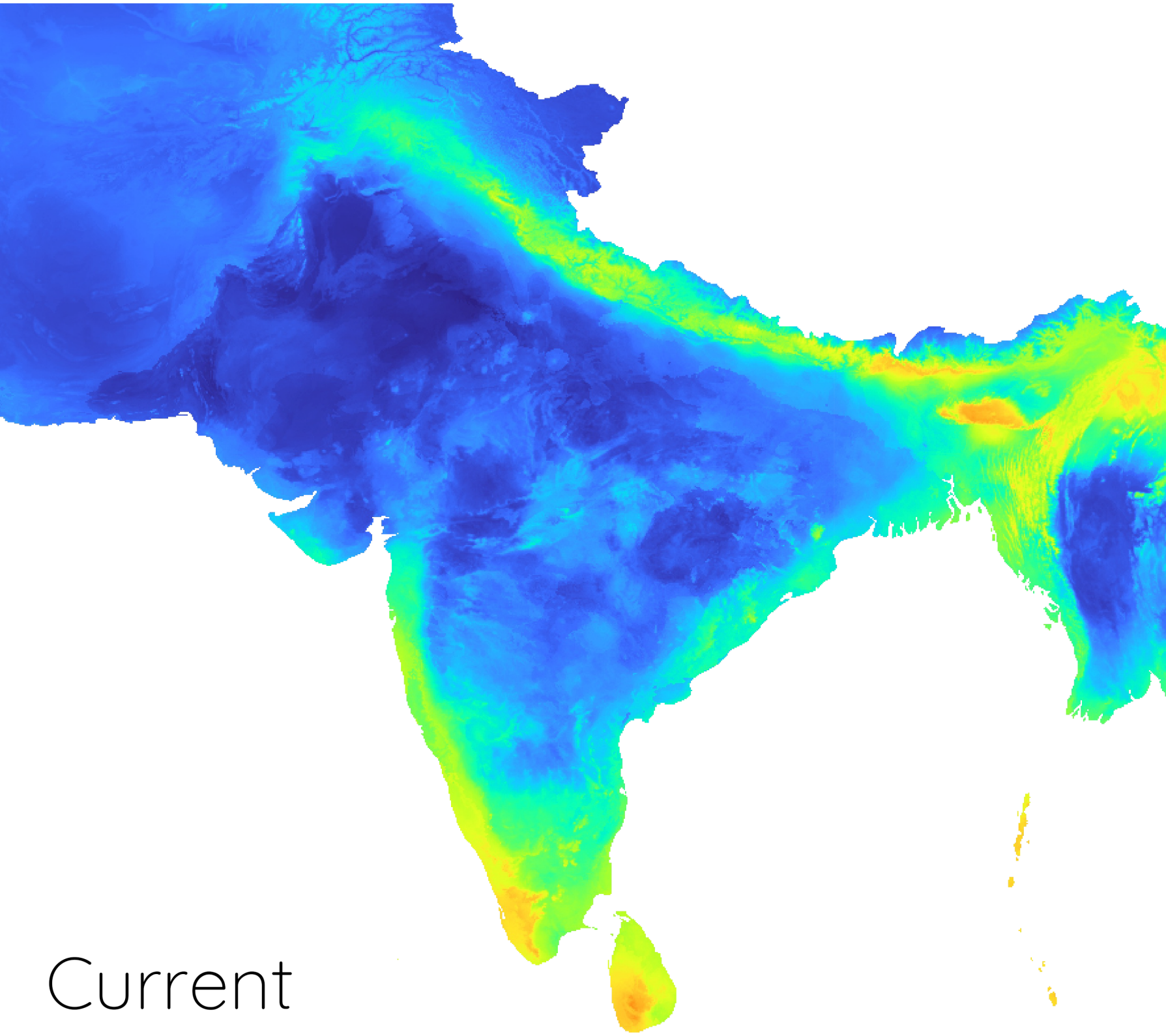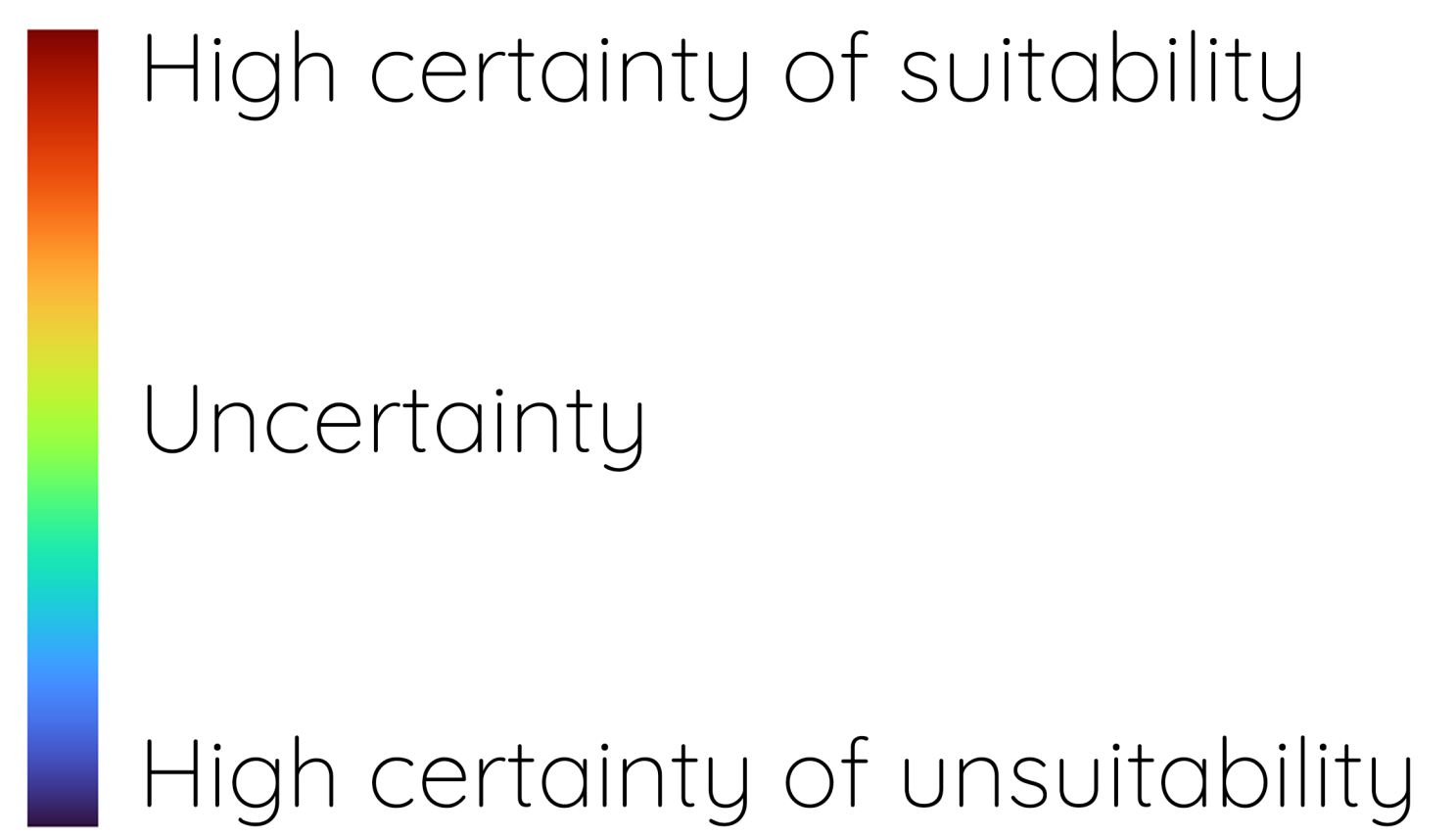

Current

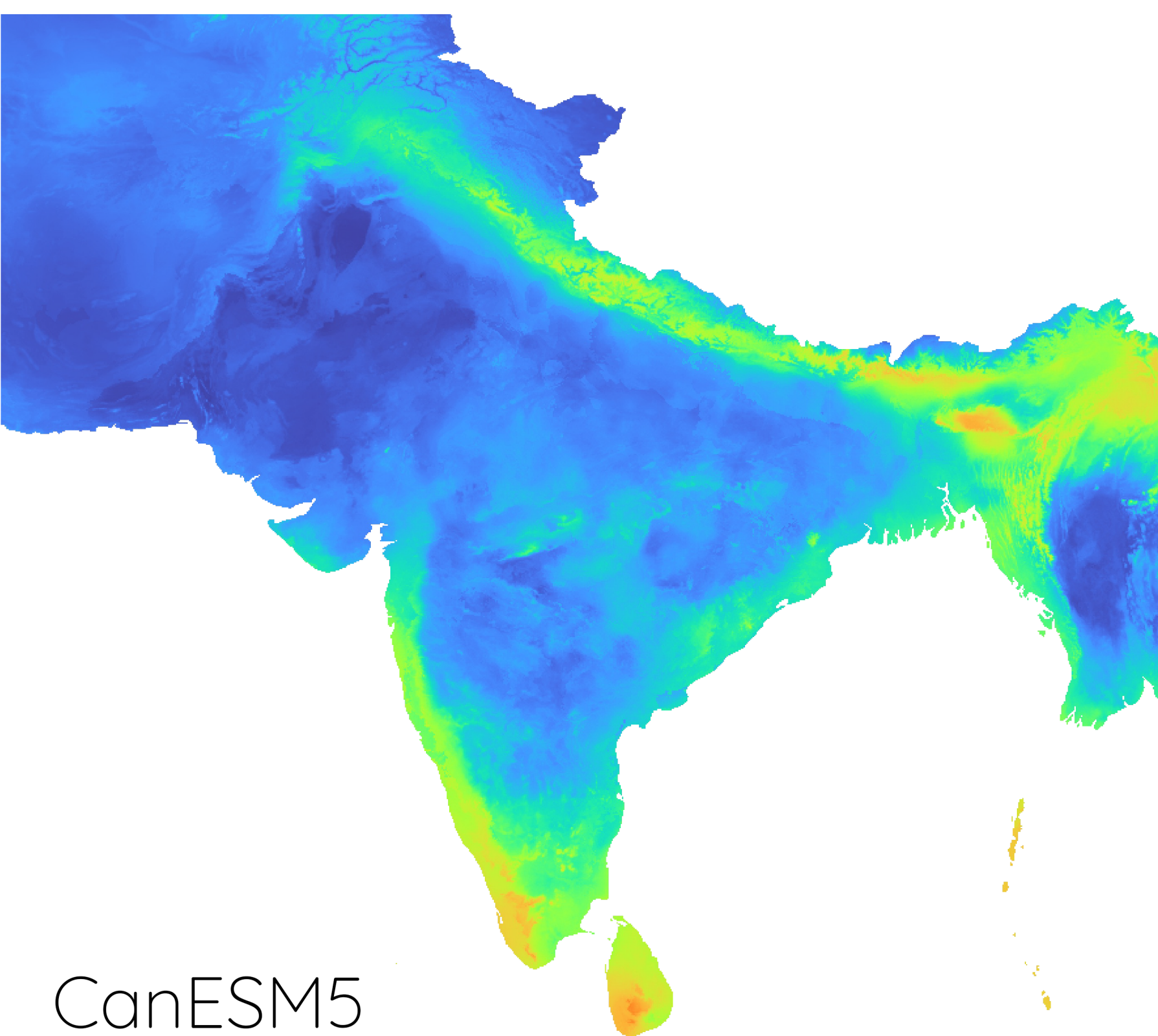

CanESM5  
SSP2-RCP4.5

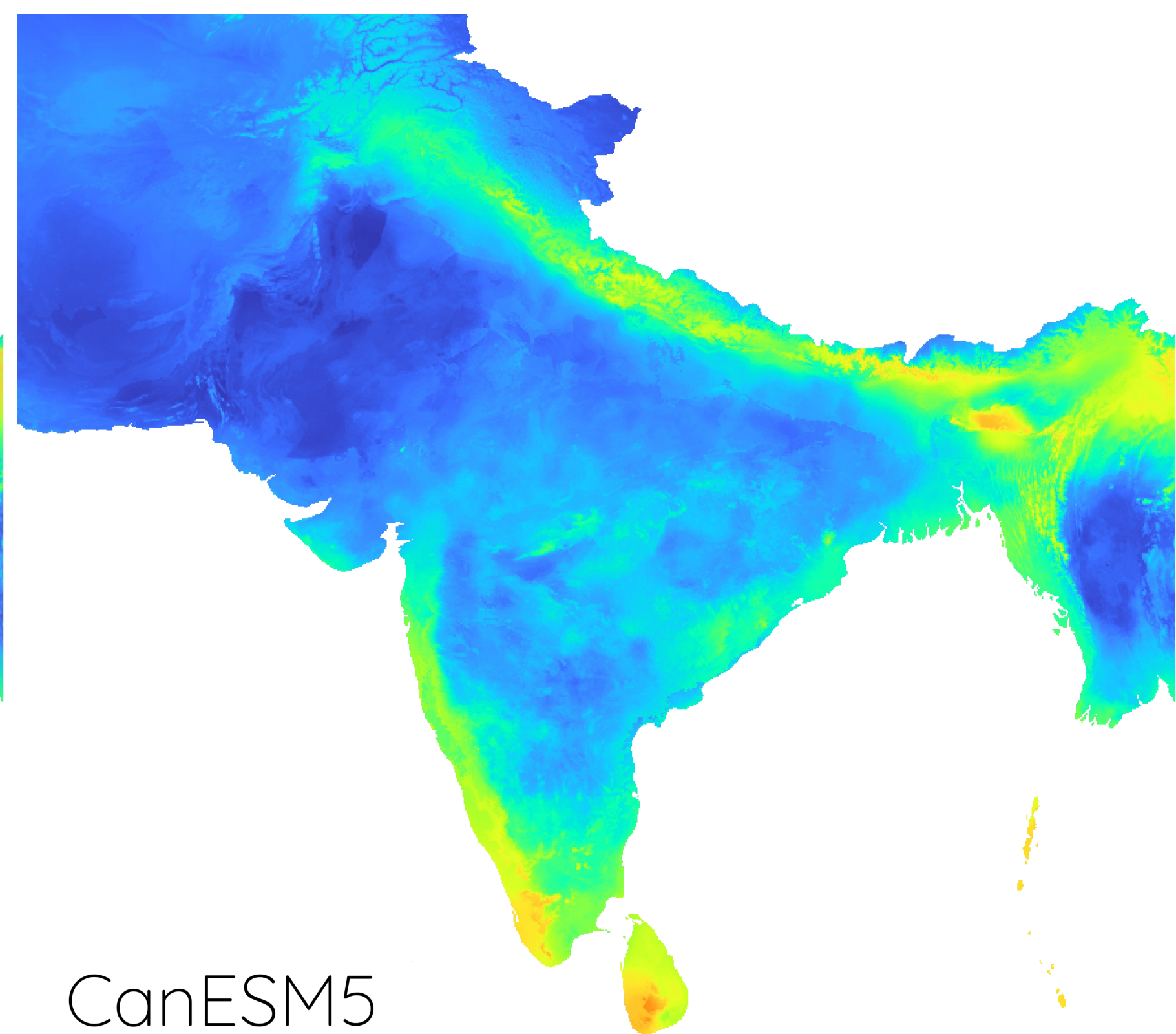

CanESM5  
SSP5-RCP8.5

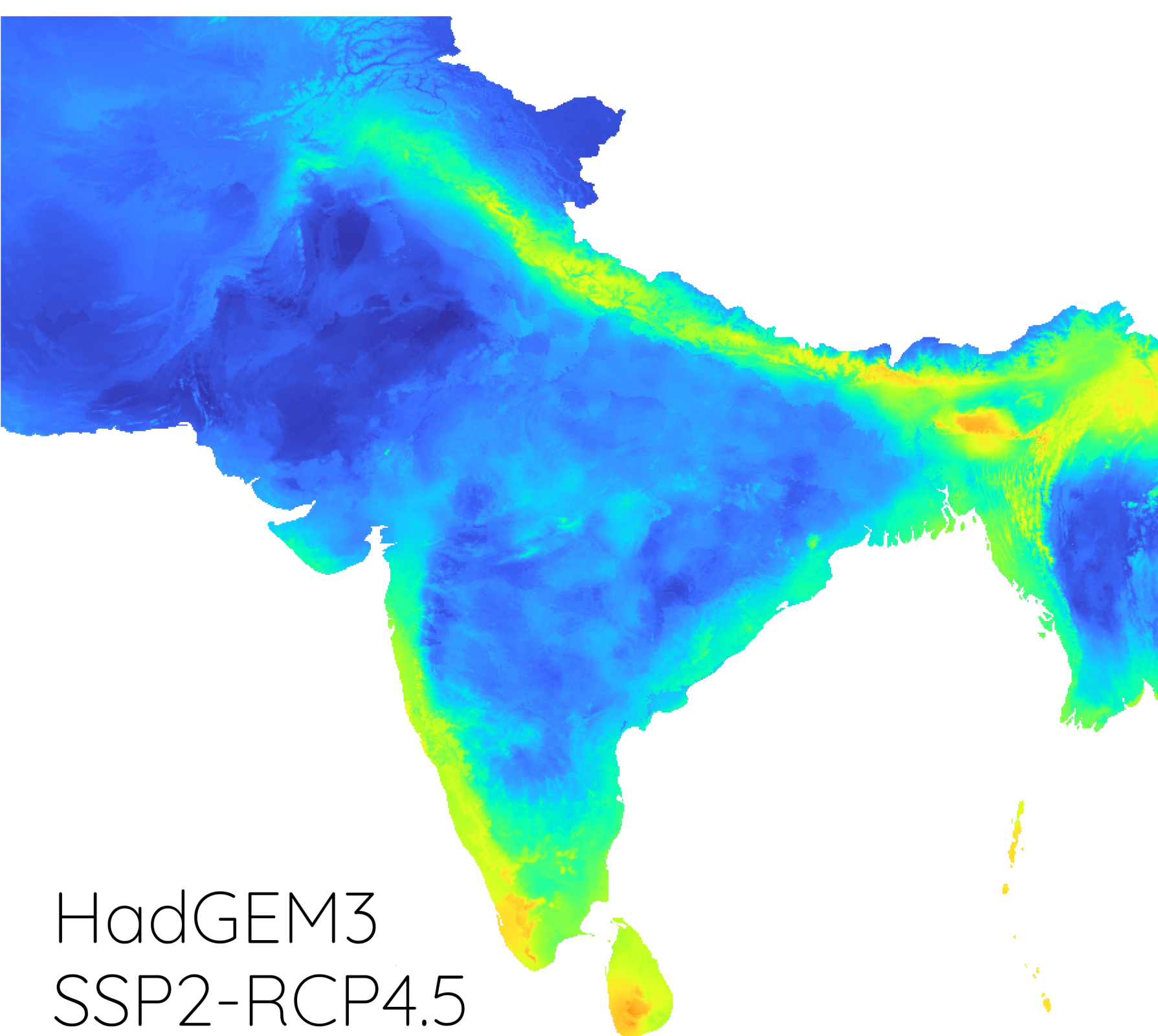

HadGEM3  
SSP2-RCP4.5

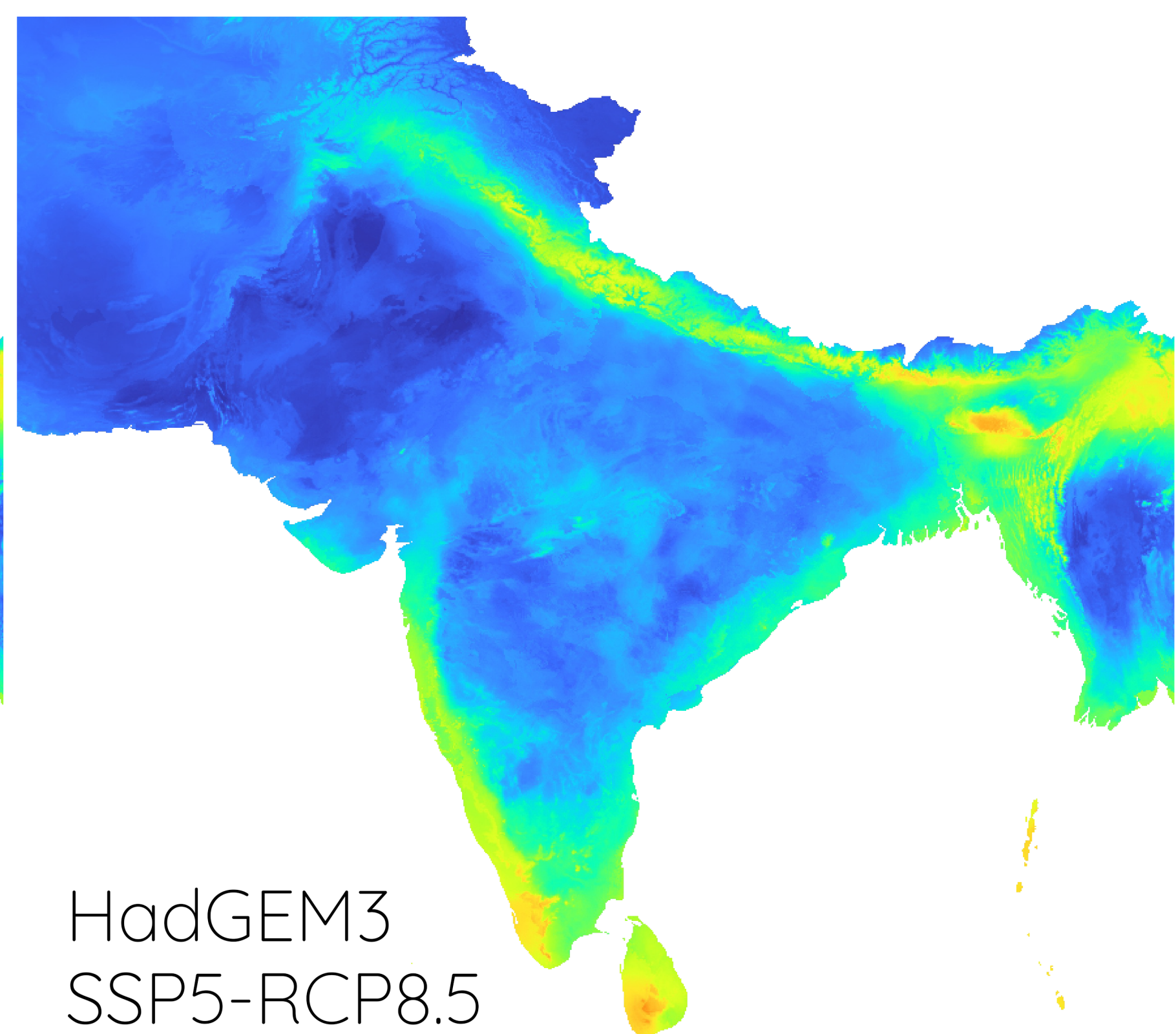

HadGEM3  
SSP5-RCP8.5

Supplement: Supplementary file 1 — Figure S1. [file ECE3-14-e11420-s002.pdf]
